# A Trip through Florida

**Published:** 1887-05

**Authors:** 


### A TRIP THROUGH FLORIDA.

*Editors Atlanta Medical and Surgical Journal:*

A recent trip through Florida, "'way down in the sunny Southland," had so many attractions for me that I have determined to give my impressions of the country to your readers.

The ancient town of Fernandina was the first place we visited. Here is much to interest the tourist. The beach here is said to be the finest on the Atlantic coast. It is over twenty miles long and furnishes one of the loveliest drives I ever saw. The spirit of improvement is manifest here on every hand. The health of the place for the past several years has been good. The present sanitary condition of the town speaks well for the authorities.

Jacksonville was the next place visited. Here one finds Florida's chief city. No one would feel that they had visited Florida unless they spent some time in this beautiful city. From Jacksonville your correspondent went to the historic city of St. Augustine. Here are many places of interest to the tourist. The old fort, built nearly three hundred years ago, is one of the chief objects of interest to the visitor. It is now filled with Indians who are confined here by general the government. Near the fort are to be seen the city gates, the remains of a wall that once encircled the old Spanish city. One place the natives take great pride in pointing out to you, unless they should find out that you are not "from the North," is the old slave market. We are confident that there never was a slave sold there. It was evidently constructed for a market, but not for the sale of slaves. From this city we returned to Jacksonville, and started south via that splendid road, the F. R. N. Just here let me say that we found on this line the most accommodating and attentive employees we ever met. Our run carried us through Baldwin, Starke, Waldo and other thriving towns until we reached Hawthorne. There we stopped for a rest. Near Hawthorne, about two miles, we found a number of beautiful homes, forming quite a village, known as Orange Lane, made up almost entirely of Georgians. Here we find more attention paid to orange culture than any place we have thus far visited. We have seen no finer groves anywhere than the groves of Messrs. Hawthorne and Johnson at this place. Dr. A. H. Shi, formerly a prominent Georgia physician, has a beautiful tract of land here with forty acres set in orange trees.

This section of Florida, in point of health, will compare favorably with the hill country in Georgia. Dr. Shi, a well-informed and reliable gentleman, informed me that there was less malaria in this section than in several of the counties of middle Georgia in which he once lived and practiced medicine.

It is my opinion that this section of Florida is destined in the near future to become the best part of the State. People can live here all the year round without inconvenience. The oranges grow here as well as in any part of the State and suffer no more from cold than those farther south.

From Citra, eight miles south of Hawthorne, more than one-third of the orange crop of Florida is shipped. Here are to be found the largest natural orange groves in the State.

At Silver Spring we found the greatest wonder we have seen in this land of—to us—many wonders. It is located at the head of the navigable waters of the Ocklawaha river. The basin of the spring covers about three acres, and it is estimated that there is an overflow of the basin of 30,000 gallons per minute. The temperature of the water is 72 degrees, and it is said that it never varies. The spring is from fifty to eighty feet deep. Notwithstanding this great depth the water is so clear that the smallest object can be seen on the bottom. Large fish are to be seen everywhere lazily swimming around in the clear water. Steamboats of considerable size run up into the spring. The outlet of the spring is a river varying in width from 100 to 40 feet, and in depth from 15 to 50 feet. One of the most fascinating trips we ever made was down this "run" on a steamboat at night.

Leaving Silver Spring, we went to Orlando and thence to Tampa. At Orlando are to be found quite a number of Georgians. It is a thriving little city and gives promise of being a place of considerable importance.

At Tampa we found another old town. For a long time it has been at a standstill, but for the past four years, since the completion of the South Florida Railroad, it has been growing, and now the new part of the city is as handsome as almost any place we saw. Here are to be found many fine orange groves.

Florida promises very little to doctors, judging by the number we find. In Georgia will be found in almost every place of three or four hundred people from two to four doctors. Such is not the case in most parts of Florida visited by your correspondent. There are fewer doctors here than anywhere we have been. The inhabitants say it is so healthy here that physicians cannot make a living. Whether this is true or not, I will not pretend to say. I know it is not true of the cities. The cities here, like the cities in Georgia, are abundantly supplied with doctors.

G.

*Tampa, Fla., April 3, 1887.*

